# Supplementary material for: Identification of photoperiod-induced specific miRNAs in the adrenal glands of Sunite sheep (Ovis aries)
Source: Front Vet Sci. 2022 Jul 22;9:888207. doi: 10.3389/fvets.2022.888207 (PMC9354845; doi:10.3389/fvets.2022.888207)
Supplement: Supplementary file 1 [file Data_Sheet_1.ZIP › Supplementary file/miRDeep2 file.zip!/miRDeep2 file/Novel_Structure/SPLP42A2/Novel_185.pdf]

Provisional ID	:	NC_019484.2_42634
Score total	:	2
Score for star read(s)	:	-1.3
Score for read counts	:	0
Score for mfe	:	1.7
Score for randfold	:	1.6
Score for cons. seed	:	
Total read count	:	146
Mature read count	:	146
Loop read count	:	0
Star read count	:	0

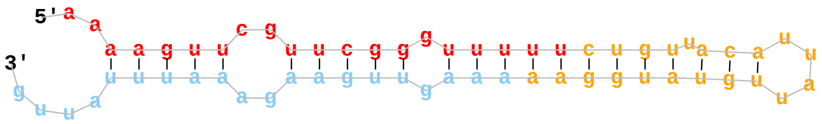

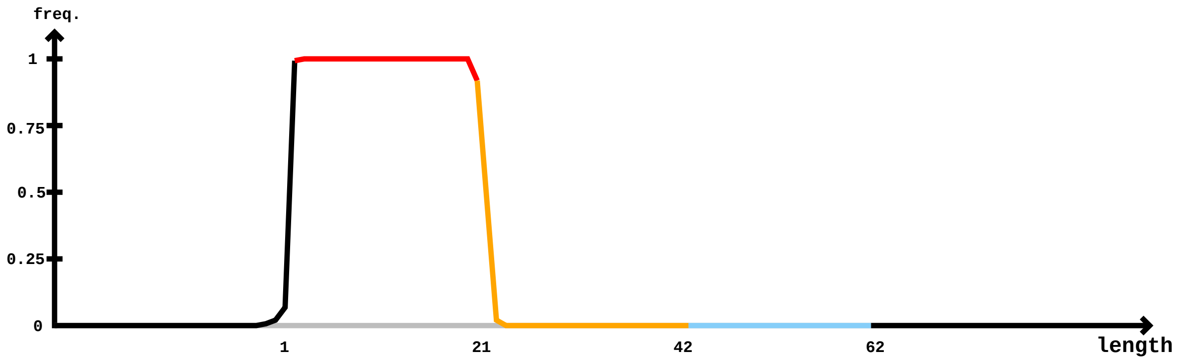

Mature Star

```
5'- aucuugaccuauuggguugguga aaaaguucguucggguuuuucuguuacauuauuguauggaaaaaguugaagaaauuuauuggccaacccaguacaacuuuggugcuuuu   -3'    exp
    ......(((((((((((((((.((.(((((..(((((.(((((((((.(((....)))))))))))).)))))..))))).)).)))))))))))))).......)))......     reads    mm    sample
    ....................ugaaaaaguucguucggguuuu......................................................................     1        0     seq
    .....................Caaaaaguucguucggguuuuu.....................................................................     2        1     seq
    ......................aaaaaguucguucggguuuu......................................................................     4        0     seq
    ......................aaaaaguucguucAgguuuuuc....................................................................     1        1     seq
    ......................aaaaaguucguucggguuuuuc....................................................................     2        0     seq
    .......................aaaaguucguucggguuuu......................................................................     7        0     seq
    .......................aaaaguucguucggguuuuu.....................................................................     126      0     seq
    .......................aaaaguucguucggguuuuucu...................................................................     3        0     seq
    ........................aaaguucguucggguuuuuc....................................................................     1        0     seq
```
